# Phyllosphere Keystone Beneficial Specialists Enhance Yield in Nutrient Deficiency‐Resistant Sorghum Cultivars

**DOI:** 10.1111/pce.70402

**Published:** 2026-01-20

**Authors:** Fangfang Li, Xiaoyan Jiao, Anqi Sun, Yong Zheng, Ju‐Pei Shen, Ji‐Zheng He, Hang‐Wei Hu

**Affiliations:** ^1^ Key Laboratory for Humid Subtropical Eco‐geographical Processes of the Ministry of Education, School of Geographical Sciences Fujian Normal University Fuzhou China; ^2^ College of Resource and Environment Shanxi Agricultural University Taiyuan China; ^3^ State Key Laboratory for Ecological Security of Regions and Cities, Ningbo Observation and Research Station, Institute of Urban Environment Chinese Academy of Sciences Xiamen China; ^4^ School of Agriculture, Food and Ecosystem Sciences, Faculty of Science The University of Melbourne Parkville Australia

**Keywords:** genotypes, keystone beneficial specialists, nutrient deficiency resistance, phyllosphere microbiomes, sorghum, yield

## Abstract

The phyllosphere, the aboveground interface between plant leaves and their microbial residents, plays a vital yet underappreciated role in crop productivity. While root‐ and soil‐associated microbiomes are well‐studied, the ecological assembly and yield‐related effects of host‐mediated phyllosphere microbial communities remain largely understudied, particularly under field conditions. This study investigates the phyllosphere microbiomes of sorghum cultivars resistant and susceptible to nutrient deficiency, focusing on how host genotype mediates microbial community assembly, keystone enrichment, and yield outcomes. The β‐diversity of phyllosphere microbiomes differs significantly between resistant and susceptible cultivars, with resistant lines also showing more modular co‐occurrence networks enriched in keystone taxa. These cultivars supported a higher abundance of keystone beneficial specialists (KBS), predominantly affiliated with Bacteroidia and Bacilli, and their abundance was positively correlated with yield. In contrast, susceptible cultivars exhibited lower and more taxonomically dispersed KBS, with a negative correlation between KBS and yield. Structural equation modeling suggested that while soil properties consistently promoted yield across cultivars, the impact of KBS on yield was genotype‐dependent. These findings reveal a host‐driven microbial mechanism linking phyllosphere composition to yield performance and highlight KBS as potential targets for microbiome‐informed breeding or foliar microbial applications to improve crop productivity in sustainable systems.

## Summary statement

We provide field‐based evidence that nutrient‐deficiency–resistant sorghum cultivars harbor a higher abundance of keystone beneficial specialists in the phyllosphere, predominantly affiliated with the classes Bacteroidia and Bacilli, compared with susceptible cultivars. These keystone taxa contribute positively to sorghum yield and therefore represent promising microbial targets for enhancing sustainable agricultural production.

## 1 Introduction

Phyllosphere, the aerial surface of plant leaves, constitutes a vast and dynamic microbial habitat, covering approximately 10^8^ km² globally and recognized as one of the largest microbial interfaces on Earth (Morris and Kinkel 2002). This extensive interface supports exceptionally high microbial colonization, with bacterial populations typically reaching 10⁶ to 10⁷ cells per cm² of leaf surface, and up to 10⁸ cells per gram of leaf tissue (Andrews and Harris 2000; Hirano and Upper 2000; Lindow and Brandl 2003; Bailey 2006; Zhu et al. 2022; Kumar et al. 2023). Despite exposure to extreme abiotic stresses such as ultraviolet radiation, high temperature, drought, and nutrient scarcity, phyllosphere microbial communities maintain remarkable diversity and structural complexity (Lindow and Brandl 2003). These microorganisms contribute to plant health by modulating hormone signaling, enhancing disease resistance, and facilitating nutrient acquisition (Stone et al. 2018; Liu et al. 2020). For example, *Aspergillus cvjetkovicii*, enriched in rice leaf microbiome, has been shown to suppress *Rhizoctonia solani* infections via small‐molecule‐mediated signaling (Fan et al. 2024). Leaf necrosis and chlorosis may occur as a consequence of disrupted microbial balance between Firmicutes and Proteobacteria in the phyllosphere (Chen et al. 2020). However, most research on plant‐associated microbiomes has focused on the rhizosphere or bulk soil (Yuan et al. 2018; Wen et al. 2022; Yue et al. 2024; Du et al. 2025), while functional phyllosphere microbiomes under field conditions remain largely underexplored.

Plant genotype (or cultivar) is a key intrinsic driver influencing the composition and assembly of microbial communities. Studies have shown that variations in leaf surface structure, volatile compound production, and immune signaling among different genotypes can selectively recruit distinct microbial taxa (Kushalappa et al. 2016; Islam et al. 2023). Phyllosphere microbial community composition of Arabidopsis, maize, and rice has been found to correlate with specific host genes, suggesting that plants may have evolved genotype‐dependent microbial selection capabilities (Balint‐Kurti et al. 2010; Redford et al. 2010; Lemke and DeSalle 2023). In wheat, benzoxazinoid compounds (e.g., DIMBOA‐Glc) produced by different host genotypes selectively promote the colonization of *Pseudomonas* (Xiang et al. 2024). Moreover, the plant innate immune system not only defends against pathogens but also plays a role in shaping beneficial plant‐microbe interactions, facilitating host‐specific colonization by commensals (Hacquard et al. 2017). Nevertheless, these studies are primarily descriptive in nature, focusing on the characterization of phyllosphere microbiome composition, leaving the microbiome‐mediated mechanisms by which host genotypes influence crop growth largely unexplored.

Plant‐associated beneficial microorganisms have long been recognized for promoting plant growth and stress resilience through diverse mechanisms such as phytohormone modulation, nitrogen fixation, and pathogen suppression (Compant et al. 2010; Müller et al. 2016; Liu et al. 2020). However, recent ecological frameworks emphasize that beneficial microbial communities are functionally heterogeneous, varying substantially in abundance, niche breadth, and ecological influence (Berry and Widder 2014; Du et al. 2025). In particular, low‐abundance taxa that are both highly connected (i.e., keystone taxa) and occupy narrow ecological niches (i.e., specialists) may disproportionately influence microbial community stability and function (Du et al. 2025). Recent studies have demonstrated that keystone microbial species can reshape the trajectory of microbiome assembly and enhance the recruitment of other functionally significant microorganisms by more than 60% (Rawstern et al. 2024). Equally critical are habitat specialists, which occupy central positions within microbial networks and play pivotal roles in maintaining network stability (Yang et al. 2024). While such taxa have been extensively studied in the rhizosphere and endosphere (Lu et al. 2025), their presence, ecological importance, and potential functional roles in the phyllosphere remain largely unknown. More importantly, current research rarely integrates network centrality, ecological specialization, and functional benefits into a unified framework, limiting our ability to identify the most influential taxa in plant‐associated microbiomes. Therefore, in this study, we propose the concept of “keystone beneficial specialists” (KBS), which refers to taxa that concurrently exhibit structural centrality, narrow niche breadth, and positive contributions to plant performance. Adopting this integrated framework not only enables a more mechanistic understanding of phyllosphere ecological processes but also significantly strengthens our capacity to identify functionally meaningful microbial groups and interpret plant‐microbe interactions.

In this study, we focused on sorghum, a major cereal crop widely cultivated in arid and semi‐arid regions for food, feed, and bioenergy production. Owing to its remarkable tolerance to drought and nutrient stress, sorghum plays a key role in ensuring agricultural productivity on marginal lands. To better understand how host genotypes influence phyllosphere microbial communities under nutrient limitation, we selected ten sorghum cultivars (five nutrient‐deficiency‐resistant and five susceptible) based on long‐term, multi‐regional field performance data, where yield stability consistently differentiated the two groups. By combining field sampling with multidimensional ecological analyses, we aimed to explore how host genotypes influence microbial community structure, functional traits, and the presence of key microbial taxa with potential roles in mediating yield variation between resistant and susceptible cultivars. Specifically, we tested the following hypotheses: (1) resistant and susceptible sorghum cultivars differentially shape phyllosphere microbial community assembly processes; and (2) resistant cultivars possess a superior genetic capacity to selectively enrich KBS in the phyllosphere, thereby establishing a more efficient microbiome that supports higher yields compared to susceptible cultivars.

## 2 Materials and Methods

### 2.1 Sample Collection

Sorghum‐associated samples were collected in September 2019 from the Dongyang Experimental Station in Shanxi Province, China (37°33′21″ N, 112°40′2″ E). The experiment was conducted using a randomized block design with each plot measuring 15 × 5 m². Ten sorghum cultivars were evaluated, including five resistant cultivars (JinZa31, FengZa4, JinFeng301, JiNiang2, LiaoZhan3) and five susceptible cultivars (JinZao5564, JinZa34, JinZa35, FenJiuLiang1, JinNuoLiang6). Resistant cultivars refer to sorghum genotypes that are capable of maintaining normal growth and yield even under nutrient‐poor soil conditions. In contrast, susceptible cultivars exhibit poor growth and reduced yield when exposed to the same low‐nutrient environments. Each cultivar was grown under two nitrogen fertilization treatments (with and without fertilization) with three biological replicates. Phosphorus and potassium were applied as diammonium phosphate and potassium sulfate, respectively, at the rate of 75 kg hm⁻² per year as base fertilizers. Nitrogen fertilizer was applied in the fertilized plots as urea at the rate of 150 kg N hm⁻² per year before sowing, while the control plots received no nitrogen fertilizer. Samples were collected from four compartments (phyllosphere, root endosphere, rhizosphere soil, and bulk soil) at the ripening stage of sorghum, resulting in a total of 240 samples.

Leaf (2–3 healthy and mature leaves per plant) were collected using sterile scissors at a standardized height of approximately 60 cm above the ground. Sampling targeted the middle functional leaves, strictly avoiding immature apical leaves and senescent basal leaves. To minimize surface contamination and physical damage, leaves were handled solely by the leaf base (collar) during cutting, ensuring the leaf surfaces were neither touched nor folded. Rhizosphere soils were collected by digging up the whole sorghum plant with a spade after removing surface debris such as stones and weeds. The roots were shaken vigorously to remove loose soil, and the soil tightly attached to the roots was collected (about 500 g per plot). Bulk soils (0–15 cm depth) were collected using a soil corer at a distance of 20 cm away from the roots and mixed thoroughly, and approximately 500 g of soil was collected for each plot. Before DNA extraction, the harvested sorghum leaf and root samples were preserved at −80°C. The soil samples were sieved through a 2‐mm mesh and divided into two portions: one was air‐dried for soil physicochemical analysis, while the other was stored at −80°C for subsequent DNA extraction.

### 2.2 Soil Physicochemical Measurement and DNA Extraction

Key soil physicochemical properties, including pH, available phosphorus (AP), soil moisture, dissolved organic carbon (DOC), ammonium nitrogen (NH₄⁺‐N), and nitrate nitrogen (NO₃⁻‐N), were measured as previously described (Sun et al. 2021). Leaf phyllosphere, root endosphere, rhizosphere soil and bulk soil samples were processed for DNA extraction using the DNeasy PowerSoil DNA Isolation Kit (Qiagen, Hilden, Germany). For phyllosphere communities, ~5 g of leaf material was agitated in phosphate‐buffered saline, sonicated, and vacuum‐filtered to collect microbial cells. Roots were surface‐sterilized (30% H₂O₂, sterile water, 70% ethanol), validated by plating rinse water on LB medium (no growth after 7 d at 30°C), and then ground under liquid nitrogen. DNA purity and yield were assessed with a NanoDrop One spectrophotometer (Thermo Fisher Scientific, Waltham, MA, USA).

### 2.3 Amplicon Sequencing and Bioinformatics

Bacterial and fungal amplicons were generated by PCR using the universal primer pair 515 F/806 R for the V3‐V4 region of the 16S rRNA gene and ITS1F/ITS2R for the fungal ITS1 region (Bates et al. 2010; Orgiazzi et al. 2012). The thermocycling protocol was initiated with a 3 min initial denaturation at 95°C, followed by 27 cycles consisting of three stages: 30 s at 95°C, 30 s at 55°C, and 45 s elongation at 72°C. A final extension step was performed at 72°C for 10 min before cooling to 4°C for reaction termination. Purified PCR products were normalized and subjected to paired‐end sequencing via the Illumina MiSeq platform (Majorbio, Shanghai, China).

Bioinformatic processing was conducted following established QIIME workflows (Caporaso et al. 2010) for sequence quality control and microbial community characterization. Bacterial community composition was determined through alignment with the SILVA reference database (Release 138, Pruesse et al. 2007), while fungal taxonomic assignment employed the UNITE dynamic dataset (Nilsson et al. 2019). To ensure analytical reliability, operational taxonomic units (OTUs) with total read counts > 20 were retained and rarefied to 18,858 and 31,053 sequences per sample for bacteria and fungi, respectively, resulting in 7690 bacterial (1159 in phyllosphere) and 1907 fungal OTUs (543 in phyllosphere). Chloroplast and mitochondrial sequences were removed from bacterial datasets prior to analysis.

### 2.4 Statistical Analyses

Statistical comparisons of alpha diversity among cultivars were conducted through one‐way ANOVA with significance threshold set at *p* < 0.05 (SPSS 25, IBM Corp., Armonk, NY, USA). To evaluate structural dissimilarities in microbial communities, beta diversity patterns were visualized through principal coordinate analysis (PCoA) computed from the Bray‐Curtis dissimilarity matrices. Multivariate community variations were statistically verified using permutational multivariate analysis of variance (PERMANOVA) with 999 permutations in R “vegan” package. In subsequent analyses, the bacterial and fungal communities were represented by their PCoA1 values. Since significant differences in bacterial and fungal diversity between cultivars were observed only in the phyllosphere, subsequent analyses focused exclusively on the phyllosphere microbiome, comprising a total of 60 samples (10 cultivars × 2 treatments × 3 biological replicates).

To assess the role of deterministic and stochastic processes in microbial community assembly, we calculated the βNTI (Beta Nearest Taxon Index) for both bacterial and fungal communities (Wen et al. 2022). A βNTI value greater than 2 or less than −2 was considered indicative of deterministic processes, while values between −2 and 2 suggested the dominance of stochastic processes. This index was calculated based on the phylogenetic turnover of microbial taxa within each plant group. Ecological processes were partitioned into deterministic (e.g., selection) and stochastic (e.g., drift, dispersal) components to assess their roles in microbial community assembly (Stegen et al. 2012). We quantified the relative importance of these processes by comparing the proportions of variance explained by each in the total community assembly. To further investigate the role of neutral processes, we fitted the Sloan neutral model to the community data, assessing the goodness of fit using R² values. The migration rate (m) was estimated from the model, which reflects the dispersal limitation or connectivity within the microbial community (Zorea et al. 2024). The neutral model fitting was performed separately for both bacterial and fungal communities associated with susceptible and resistant cultivars.

Niche breadth was assessed for both bacterial and fungal communities based on the Levins’ niche breadth index, which quantifies the degree of resource or habitat utilization (Du et al. 2025). Higher values indicate generalist species capable of thriving in a wide range of conditions, while lower values indicate specialist species adapted to narrower niches. Niche breadth was calculated using the “EcolUtils” package in R, and the statistical significance of niche classification was assessed via 1,000 permutations; taxa falling above or below the 95% confidence intervals were designated as generalists or specialists, and those within the confidence interval were classified as neutral taxa. Comparisons of niche breadth between susceptible and resistant cultivars were conducted using *t*‐tests for both bacterial and fungal communities. Additionally, niche breadth across the four subgroups (CS = Control + Susceptible; CR = Control + Resistant; FS = Fertilized + Susceptible; FR = Fertilized + Resistant) was analyzed using one‐way ANOVA, followed by least significant difference (LSD) post hoc tests to identify significant differences in community.

### 2.5 Co‐Occurrence Network Construction and Analysis

Separate co‐occurrence networks were constructed for susceptible and resistant cultivars to examine how specialist and generalist taxa within both bacterial and fungal communities interacted in each network. Spearman's correlation coefficients were calculated for all possible OTU pairs within each group. Significant correlations (|R | > 0.6, *p* < 0.01) were retained and visualized as networks using the interactive platform Gephi (0.9.2). Network topological parameters, including node degree and betweenness centrality, were also calculated in Gephi to evaluate the connectivity and relative importance of each node within the network.

Microbial ecological networks were analyzed using a topological role framework based on within‐module connectivity (Zi) and among‐module connectivity (Pi) (Nie et al. 2021). Nodes with Zi > 2.5 were classified as module hubs, those with Pi > 0.62 as connectors, and those fulfilling both criteria as network hubs. Module hubs, connectors, and network hubs were defined as keystone taxa. This classification was performed using the “ggClusterNet” and “igraph” packages in R. The relative distribution of keystone taxa across major phyla and ecological roles (specialist, neutral) was visualized using chord diagrams generated with the “circlize” package. Linear regression models were used to examine the relationship between keystone and key topological attributes (degree, betweenness centrality).

Since the majority of keystone specialists identified in this study were bacteria, subsequent analyses focused on bacterial taxa. Keystone beneficial specialists (KBS) were defined using a stepwise screening framework, whereby taxa were required to simultaneously meet three criteria: (i) identification as keystone species (including module hubs, connectors, or network hubs) based on the Zi–Pi topological analysis; (ii) classification as specialists according to the Levins’ niche breadth index combined with the null model analysis; and (iii) annotation as potentially beneficial bacteria based on the PBB database (Li et al. 2023). It should be noted that this database was primarily developed from root‐ and soil‐associated microbial taxa, and thus the functional categories derived from it may not fully represent the ecological roles of microorganisms in the phyllosphere. Therefore, the “beneficial” designation used here should be interpreted as a functional inference based on available evidence rather than a definitive characterization of phyllosphere activity. Structural equation modeling (SEM) was conducted using Amos Graphics v22 (IBM Corp., Armonk, NY, USA) to evaluate the direct and indirect effects of microbial communities (represented by PCoA1 scores), keystone taxa (identified as module hubs, connectors, and network hubs via the Zi‐Pi analysis), KBS, and soil properties (represented by the PCoA1 axis of soil physicochemical variables, including pH, AP, soil moisture, DOC, NH_4_^+^‐N and NO_3_^‐^‐N) on sorghum yield in both susceptible and resistant cultivars. The initial hypothetical model was based on ecological knowledge and prior findings, specifying paths between microbial diversity, keystone taxa abundance, soil physicochemical properties, and crop yield. Model fit was assessed using standard goodness‐of‐fit indices, including the Chi‐square statistic, goodness‐of‐fit index (GFI), and Root Mean Square Error of Approximation (RMSEA). Path coefficients were estimated using maximum likelihood estimation.

## 3 Results

### 3.1 Microbial Diversity and Community Composition Across Different Compartments of Sorghum

**Figure 1:**
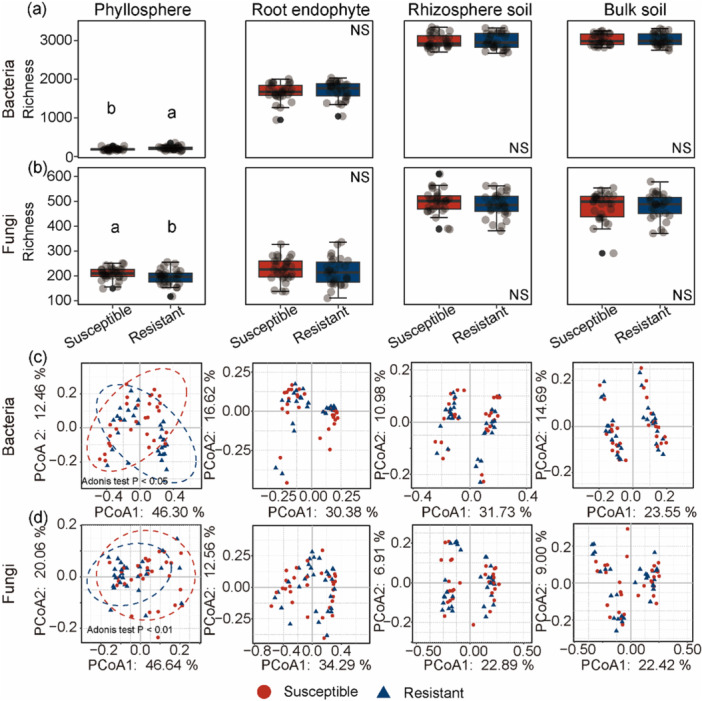
Differences in bacterial and fungal diversity across compartments between susceptible and resistant cultivars. Bacterial (a) and fungal (b) OTU richness across different plant‐associated compartments—phyllosphere, root endophytes, rhizosphere soil, and bulk soil—between susceptible and resistant cultivars. Different letters denote significant differences (*p* < 0.05) between susceptible and resistant cultivars. NS = not significant (*p* ≥ 0.05). Community structure differences were visualized via principal coordinate analysis (PCoA) using the Bray–Curtis dissimilarity for bacteria (c) and fungi (d), with significance assessed by Adonis testing.

**Figure 2:**
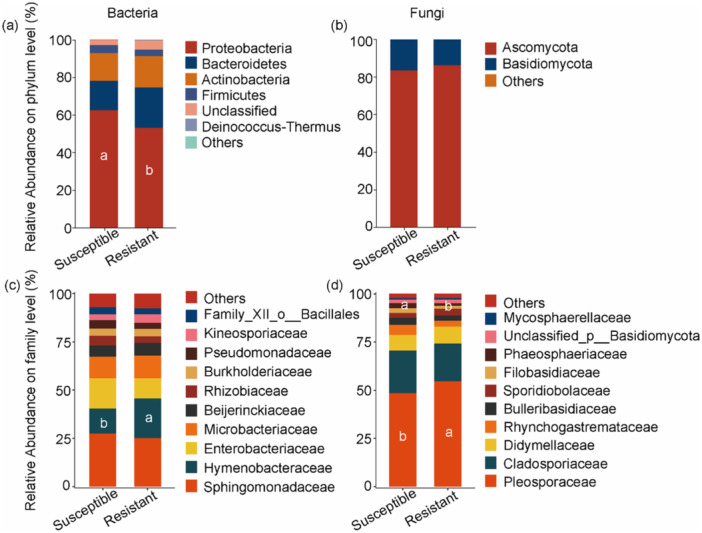
Community composition of bacteria and fungi at the phylum (a−b) and family (c−d) levels in susceptible and resistant cultivars. Different letters indicate statistically significant differences (*p* < 0.05) in the same microbial taxon between susceptible and resistant cultivars. [Color figure can be viewed at wileyonlinelibrary.com]

Among the four compartments, significant differences in both bacterial (Figure 1a) and fungal (Figure 1b) richness, as well as community structure (Figure 1c,d), were observed between susceptible and resistant cultivars only in the phyllosphere (*p* < 0.05). In other compartments, cultivars had no significant effect on the diversity of bacterial or fungal communities. In the phyllosphere, significant differences in microbial community composition were observed between the two cultivar types at both the phylum (Figure 2a,b) and family (Figure 2c,d) levels. Significant differences were observed in the phyllosphere bacterial phylum Proteobacteria and the family *Hymenobacteraceae* between susceptible and resistant cultivars. Similarly, the phyllosphere fungal families *Pleosporaceae* and *Phaeosphaeriaceae* differed significantly between the two cultivars (Figure 2a–d). To further elucidate the ecological mechanisms of these differences, subsequent analyses focused specifically on the phyllosphere microbiome.

### 3.2 Ecological Traits of Phyllosphere Microbiome Across Cultivars

**Figure 3:**
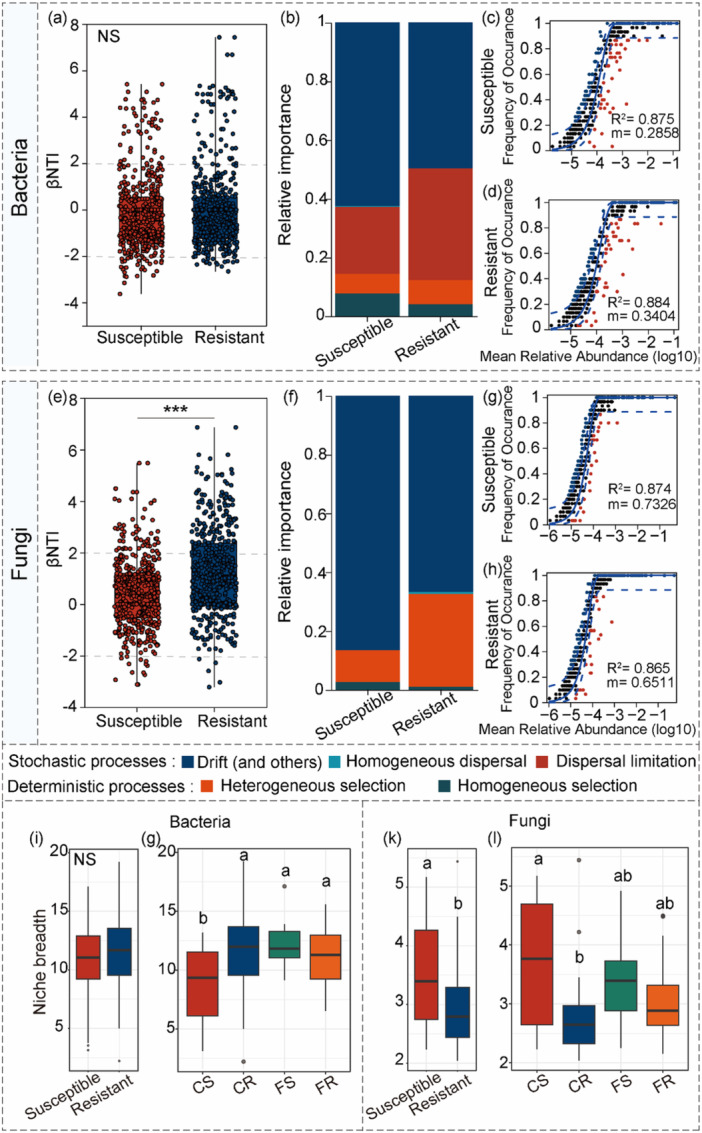
Community assembly mechanisms and niche breadth of bacterial and fungal communities in susceptible and resistant cultivars. The distributions of Beta‐Nearest Taxon Index (βNTI) for bacterial (a) and fungal (e) communities between susceptible and resistant cultivars. Asterisks denote significant differences in proportions as determined by *t*‐tests (*p < 0.05, **p < 0.01, ***p < 0.001). Deterministic processes were categorized based on βNTI thresholds: homogeneous selection (βNTI < − 2), heterogeneous selection (βNTI > 2), and Stochastic processes (−2 ≤ βNTI ≤ 2). Contribution of bacterial (b) and fungal (f) community assembly processes in susceptible and resistant cultivars. Neutral community model (NCM) fitting for bacterial and fungal communities between susceptible and resistant sorghum cultivars. Predicted OTUoccurrence frequencies in soil microbial communities are shown for bacterial (c–d) and fungal (g–h) assemblages. Solid blue lines represent NCM best‐fit curves, with dashed blue lines indicating 95% confidence intervals around the model prediction. Model fit is quantified by R² (coefficient of determination) and microbial migration rate *m*. Niche breadth of bacterial (i, g) and fungal (k, l) communities under different management measures and cultivars. In (i, k), *t*‐test was used to compare susceptible and resistant cultivars. In (g, l) differences among the four treatment groups (CS = Control + Susceptible; CR= Control + Resistant; FS=Fertilized + Susceptible; FR= Fertilized + Resistant) were assessed using ANOVA followed by LSD test (*p* < 0.05). Different letters indicate significant differences; NS: not significant. [Color figure can be viewed at wileyonlinelibrary.com]

The βNTI analysis revealed that stochastic processes dominated both bacterial and fungal communities in the phyllosphere across susceptible and resistant cultivars (Figure 3a–h). For bacterial communities, cultivar type did not have a significant impact on community assembly or niche breadth (Figure 3a,i), with stochastic processes—primarily drift—dominating community assembly (> 80% relative importance, Figure 3b). For fungal communities (Figure 3e), resistant cultivars exhibited significantly higher βNTI values (*p* < 0.001). Although stochastic processes dominated both groups, this increase in βNTI signifies a distinct shift toward deterministic processes, specifically driven by heterogeneous selection (Figure 3f). This shift toward determinism in resistant cultivars was further supported by the neutral model analysis, which revealed lower migration rates (m) in resistant versus susceptible cultivars (Figure 3g,h). Analysis of microbial niche breadth demonstrated a significant reduction in fungal niche width in resistant cultivars, reflecting an enrichment of specialist taxa, thus providing additional evidence for a transition toward deterministic, heterogeneously selected community assembly (Figure 3k,l).

### 3.3 Differences in Microbial Co‐Occurrence Networks Between Susceptible and Resistant Cultivars

**Figure 4:**
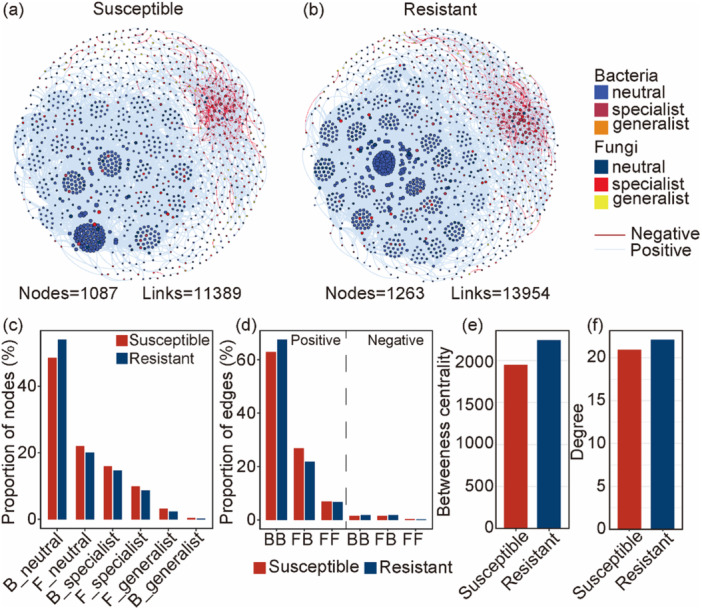
Co‐occurrence network characteristics of microbial communities associated with susceptible and resistant cultivars. Panels (a) and (b) show the microbial networks for susceptible and resistant cultivars, respectively. Edges indicate significant correlations: red = negative, blue = positive. (c) Proportions of different ecological categories of nodes. (d) Proportions of positive and negative correlations between bacteria–bacteria (BB), fungi–bacteria (FB), and fungi–fungi (FF). (e) Comparison of betweenness centrality between networks. (f) Comparison of average degree between networks. Red and blue bars represent susceptible and resistant cultivars, respectively. [Color figure can be viewed at wileyonlinelibrary.com]

Co‐occurrence network analysis revealed distinct structural and compositional differences in microbial interactions between the susceptible and resistant cultivars. Resistant cultivars exhibited a more complex and connected network, with a higher number of nodes (1263 vs. 1087) and edges (13,954 vs. 11,389) compared to susceptible cultivars (Figure 4a,b). In both networks, bacterial neutral taxa comprised the largest proportion of nodes, and their relative abundance was higher in resistant cultivars (Figure 4c). Fungal and bacterial specialists were also more prevalent in resistant cultivars, while generalists were rare across both networks (Figure 4c). Positive associations overwhelmingly dominated the networks in both groups. Resistant cultivars exhibited a higher proportion of positive bacterial–bacterial (BB) interactions but a lower proportion of positive fungal–bacterial (FB) interactions, while positive and negative fungal–fungal (FF) interactions remained virtually unchanged between the two cultivars (Figure 4d). Topological features further highlighted the enhanced connectivity in the resistant cultivar's network. Betweenness centrality, a measure of a node's importance in connecting other nodes, was higher in the resistant network (Figure 4e), as was the average degree, indicating more connections per node (Figure 4f). Collectively, these findings suggest that the microbial community associated with the resistant cultivars is more complex, interactive, and potentially more robust against perturbations.

### 3.4 Topological Roles and Taxonomic Identity of Keystone Taxa Differ Between Resistant and Susceptible Cultivars

**Figure 5:**
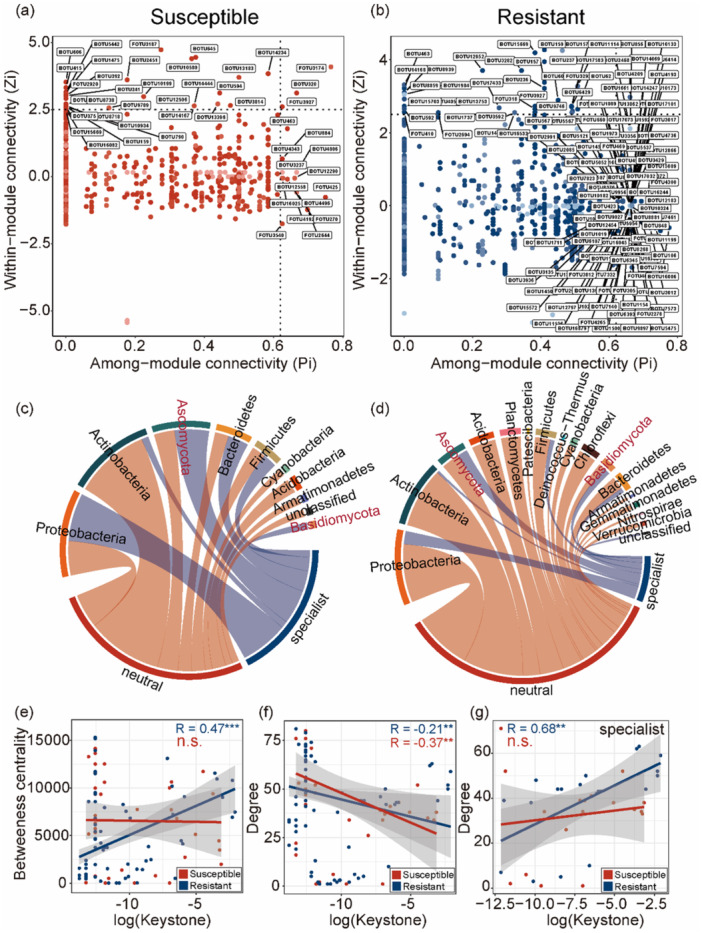
Topological roles and taxonomic identities of keystone taxa in microbial networks of susceptible and resistant cultivars. The within‐module (Zi) and among‐module (Pi) connectivity of nodes for susceptible (a) and resistant (b) cultivars. Chord diagrams showing the distribution of neutral and specialist taxa across different bacterial and fungal phyla in susceptible (c) and resistant (d) cultivars. Relationships between log‐transformed keystone scores and network metrics: (e) betweenness centrality and (f) degree, based on all identified keystone taxa (Zi > 2.5 or Pi > 0.62). (g) Relationship between log‐transformed keystone scores and degree for specialist keystone taxa. Solid lines represent linear regressions for susceptible (red) and resistant (blue) cultivars. Shaded areas indicate 95% confidence intervals. R values denote Pearson correlation coefficients. Asterisks indicate significance levels: *p < 0.05, **p < 0.01, ***p < 0.001; n.s., not significant. [Color figure can be viewed at wileyonlinelibrary.com]

Network role classification based on within‐module connectivity (Zi) and among‐module connectivity (Pi) revealed distinct patterns in the two cultivars (Figure 5a,b). In the resistant network, more keystone taxa (i.e., module hubs, connectors, and network hubs) were identified compared to the susceptible network, indicating enhanced complexity and modularity. Chord diagrams (Figure 5c,d) showed that keystone taxa were primarily affiliated with Proteobacteria, Actinobacteria, and Ascomycota in both networks. However, resistant cultivars harbored a greater diversity of keystone phyla, including Planctomycetota, Verrucomicrobia, and Chloroflexi.

Correlation analyses revealed a significantly positive relationship between log‐transformed keystone index and betweenness centrality in resistant cultivars, but not in susceptible cultivars (Figure 5e). Degree was negatively correlated with keystone index in susceptible and resistant cultivars, but showed a weaker correlation in resistant cultivars (Figure 5f). Among specialist taxa, a strong positive correlation between keystone and degree was observed only in resistant cultivars (Figure 5g), highlighting the potential regulatory role of specialists in resistant cultivars.

**Figure 6:**
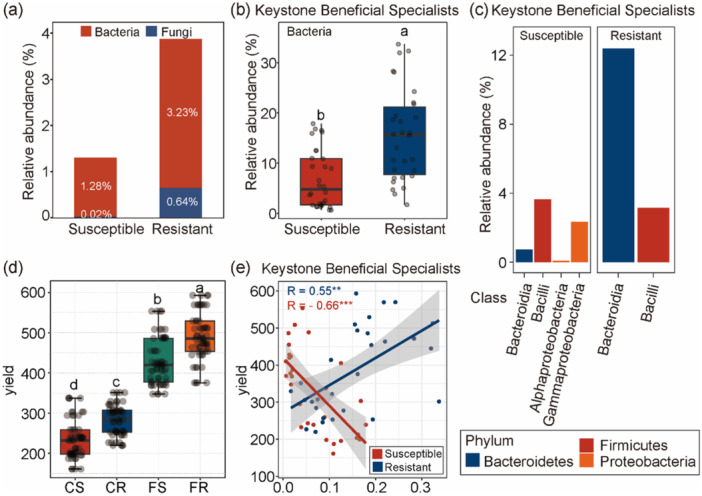
Relative abundance and functional implications of keystone taxa and keystone beneficial specialist (KBS) in susceptible and resistant cultivars. Relative abundance of keystone taxa (a) and of KBS (b) in susceptible and resistant cultivars. Different letters indicate statistically significant differences (*p* < 0.05). Taxonomic composition of beneficial bacteria within specialist keystone taxa at the class level. The bars represent the average relative abundance (%) of each bacterial class, colored by phylum. (d) Yield differences among four groups: CS = Control + Susceptible; CR = Control + Resistant; FS = Fertilized + Susceptible; FR = Fertilized + Resistant. Different letters indicate statistically significant differences (*p* < 0.05). (e) Regression analysis showing the correlation between the abundance of KBS and yield in susceptible (red) and resistant (blue) cultivars. R values represent Pearson correlation coefficients, with significance indicated by asterisks (**p < 0.01, ***p < 0.001). [Color figure can be viewed at wileyonlinelibrary.com]

Bacterial taxa consistently accounted for a larger proportion of specialist keystone taxa than fungal taxa in both susceptible and resistant cultivars. Specifically, the relative abundance of bacterial keystone specialists was 1.28% in susceptible cultivars and 3.23% in resistant cultivars, whereas fungal counterparts contributed only 0.02% and 0.64%, respectively (Figure 6a). To further identify functionally important members among the keystone specialists, we specifically focused on keystone beneficial specialists (KBS). The relative abundance of KBS was significantly higher in resistant cultivars than in susceptible ones (Figure 6b), the taxonomic composition of KBS differed markedly between susceptible and resistant cultivars. In susceptible cultivars, KBS were present at low relative abundances, with *Bacilli* (Firmicutes) being the dominant class, followed by minor contributions from *Gammaproteobacteria* (Proteobacteria), *Bacteroidia* (Bacteroidetes), *Alphaproteobacteria* (Proteobacteria). In contrast, resistant cultivars exhibited a substantially higher proportion of KBS, dominated primarily by *Bacteroidia* and *Bacilli* (Figure 6c).

### 3.5 Driving Factors Influencing the Sorghum Yield

Overall sorghum yield tended to be higher in resistant than in susceptible cultivars (Figure 6d), and yield increased progressively from CS to CR, followed by FS, with FR exhibiting the highest yield. Pairwise comparisons indicated that all four treatments differed significantly from each other (Figure 6d). A significant negative relationship was found between KBS abundance and yield in susceptible cultivars, while the relationship was significantly positive in resistant cultivars (Figure 6e).

**Figure 7:**
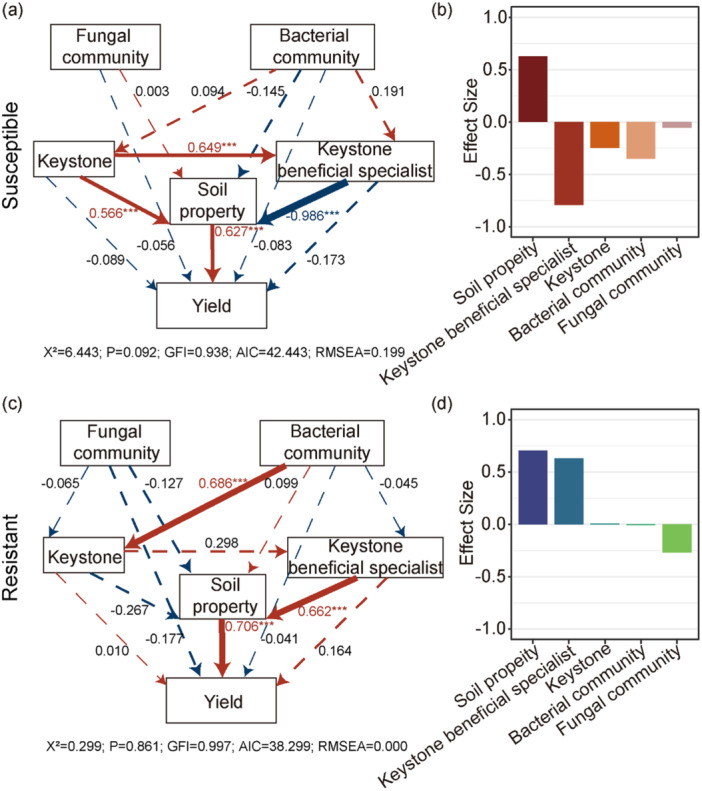
Structural equation models (SEMs) illustrating the relationships between microbial communities, keystone taxa, soil properties, and sorghum yield in (a) susceptible and (c) resistant cultivars. Red and blue arrows indicate positive and negative relationships, respectively, with line thickness representing the strength of the relationship. Significant paths are marked with asterisks (*p < 0.05, **p < 0.01, ***p < 0.001). Panels (b) and (d) show the total effect sizes of different factors on yield in susceptible and resistant cultivars, respectively. [Color figure can be viewed at wileyonlinelibrary.com]

To elucidate the mechanisms underlying yield variation between susceptible and resistant sorghum cultivars, we constructed SEMs incorporating microbial community composition, keystone taxa, KBS, soil properties, and yield (Figure 7). In both susceptible and resistant cultivars (Figure 7a–d), soil properties and KBS were the primary factors associated with sorghum yield. While soil properties had a consistently positive association with yield across both cultivar types, the impact of KBS diverged sharply: they were significantly negatively associated with yield in susceptible cultivars but showed a significantly positive association in resistant cultivars. In susceptible cultivars, the negative association of KBS outweighed the positive contribution of soil properties (Figure 7a–d). In contrast, in resistant cultivars, the positive association of these keystone microbes not only enhanced yield but also helped explain the overall yield advantage observed in this group.

## 4 Discussion

Phyllosphere microbiomes are shaped not only by external factors such as climatic conditions and microbial dispersal (Zhu et al. 2022), but also by intrinsic plant traits encoded by the host genome (Massoni et al. 2020; Yue et al. 2024). Differences in plant genotypes can result in distinct microbial communities with varying levels of diversity, network stability, and functional potential (Rodríguez‐Blanco et al. 2015; Yue et al. 2024). These effects are particularly pronounced under stress conditions, where genotype‐specific microbiomes may enhance plant resilience through ecological filtering or keystone taxa enrichment (Lebeis et al. 2015; Finkel et al. 2017; Banerjee et al. 2018). However, the extent to which host genotype influences the ecological assembly of phyllosphere microbiome and whether such influences translate into yield differences remain largely unresolved. As a critical step toward harnessing the potential of phyllosphere microbiome for improving crop performance, it is essential to unravel how host genetic background regulates microbial community structure, key microbial taxa, and their functional consequences. In this study, we examined how different sorghum genotypes influence phyllosphere microbial assembly, network architecture, and the presence of keystone beneficial specialists. Our findings provide novel insights into the genotype–microbiome–productivity axis, with implications for incorporating phyllosphere microbiome management into future crop breeding and sustainable agriculture strategies.

Resistant and susceptible sorghum cultivars exhibited markedly different phyllosphere microbial assemblages, suggesting that host genotype acts as a key ecological filter (Gong and Xin 2020). Previous studies have proposed that genotype‐dependent variation in the microbiome is primarily driven by host‐mediated ecological filtering rather than by environmental factors, especially when plants are cultivated under similar external conditions (Balint‐Kurti et al. 2010; Redford et al. 2010; Wagner et al. 2016). As demonstrated in Arabidopsis and maize (Balint‐Kurti et al. 2010; Bodenhausen et al. 2013), genotype‐dependent variation in leaf surface traits (such as cuticle thickness and trichome density) in sorghum likely shapes unique microbial colonization environments by influencing leaf surface chemistry, exudate profiles, or immune signaling pathways (Hacquard et al. 2017; Islam et al. 2023; Fan et al. 2024). In our study, resistant cultivars exhibited narrower fungal niche breadth and a shift from stochastic to more deterministic community assembly processes. This deterministic process is typically driven by host traits such as specific cuticle composition, volatile organic compounds, and immune signaling, which have been shown to selectively enrich particular microbial taxa on leaf surfaces (Lebeis et al. 2015; Trivedi et al. 2020). Consequently, resistant sorghum genotypes likely preferentially enrich microbial taxa better adapted to their specific phyllosphere microenvironments through this host‐specific selection. In contrast, susceptible cultivars tend to harbor communities assembled with greater randomness and reduced ecological coherence. Co‐occurrence network analysis further reinforces this genotype‐driven differentiation. Resistant cultivars exhibited more complex and modular networks enriched with keystone specialists, primarily affiliated with Proteobacteria and Actinobacteria. Such keystone taxa are crucial for maintaining structural stability and ecological function across microbial ecosystems (Berry and Widder 2014), suggesting that these phyllosphere specialists may play similar roles in aerial habitats. Their enrichment reflects not only a more stable and hierarchically structured community but also highlights the role of host genotype in fostering ecologically coherent phyllosphere microbiome.

We found a distinction between keystone taxa and KBS, referring to the beneficial bacterial taxa within the keystone specialists that may contribute to host performance. Our findings show that the relative abundance of KBS was significantly higher in resistant cultivars and positively correlated with yield, but in susceptible cultivars, KBS abundance was negatively correlated with yield. Previous studies on plant‐associated microbiomes have shown that when host immune regulation is compromised or suboptimal, microbial communities, even those enriched with beneficial traits, can become ecologically unstable or dysbiotic, ultimately hindering host performance (Panke‐Buisse et al. 2015; Zaneveld et al. 2017). This aligns with the “Anna Karenina principle”, which posits that stressed or genetically susceptible hosts are more likely to harbor disordered microbiomes with maladaptive effects (Zaneveld et al. 2017). Similar host–microbe incompatibility has been observed in Arabidopsis and maize, where bacterial strains that promote growth in one genotype show neutral or even negative effects in another due to differences in immune signaling or metabolic compatibility (Panke‐Buisse et al. 2015; Vogel et al. 2016; Finkel et al. 2017). These findings together suggest that the negative KBS–yield correlation in susceptible cultivars may arise from ineffective host‐mediated integration of beneficial microbes, leading to unintended ecological consequences.

The KBS communities in resistant cultivars were primarily composed of two dominant bacterial classes, Bacteroidia and Bacilli. Members of Bacilli, especially species within *Bacillus subtilis* and *Bacillus amyloliquefaciens*, are well‐known producers of antimicrobial compounds such as surfactin, fengycin, and iturin, which can suppress foliar and soil‐borne pathogens (Hiradate et al. 2002; Yu et al. 2002; Ongena and Jacques 2008; Arnaouteli et al. 2021). Bacteroidia are recognized for their strong capabilities in polysaccharide degradation and involvement in complex carbon cycling, contributing to microbial nutrient exchange on plant surfaces (Thomas et al. 2011; Grondin et al. 2017). The taxonomic convergence of KBS may indicate an ecologically optimized assembly characterized by high functional complementarity and low redundancy, an architecture shown to enhance microbial community stability and efficiency under environmental constraints (Banerjee et al. 2018). Such targeted microbial assemblages may allow resistant cultivars to harness phyllosphere microbes more effectively to support yield, echoing host–microbe co‐evolutionary dynamics proposed in root systems (Trivedi et al. 2020). In contrast, susceptible cultivars not only hosted fewer KBS, but those present were taxonomically diverse with notably high levels of Gammaproteobacteria, a group frequently associated with phyllosphere dysbiosis under stress. For example, in drought‐stressed forage grasses, Gammaproteobacteria increased significantly and correlated with signs of leaf microbial community imbalance and reduced plant productivity (Bechtold et al. 2021). Furthermore, Arabidopsis mutants defective in immune signaling exhibited elevated Proteobacteria in leaf communities, leading to dysbiotic leaf symptoms (Bodenhausen et al. 2013). These findings suggest that susceptible cultivars may be unable to filter out potentially destabilizing taxa, resulting in maladaptive microbial configurations despite their “beneficial” potential (Chen et al. 2020; Bechtold et al. 2021). This may reflect weak host filtering capacity, contributing to the maladaptive yield effects observed in our study.

Further supporting these interpretations, our SEMs revealed that while soil physicochemical properties exerted a consistently positively association with yield across both resistant and susceptible cultivars, the effects of KBS abundance on yield were genotype‐specific and directionally aligned with yield outcomes. In resistant cultivars, higher KBS abundance was associated with higher yield, aligning with their overall higher productivity. In contrast, in susceptible cultivars, KBS abundance showed a negative association with yield, which corresponded to a weaker yield response despite favorable soil conditions. This genotype‐specific directionality aligns with previous findings that plant genetic background critically shapes the outcome of microbial colonization, with beneficial taxa exerting contrasting effects depending on host immune competence and metabolic compatibility (Panke‐Buisse et al. 2015; Finkel et al. 2017).

## 5 Conclusions

Our study reveals that sorghum host genotype plays a critical role in shaping phyllosphere microbiome assembly, particularly through the selective enrichment of keystone beneficial specialists. Resistant cultivars support higher KBS abundance, taxonomically dominated by Bacilli and Bacteroidia, which are correlated positively with yield. In contrast, susceptible cultivars exhibited lower KBS levels and even negative KBS–yield associations, likely due to ineffective host filtering and microbiome integration. These results provide new evidence that sorghum cultivars differ significantly in how they structure phyllosphere microbial communities. Furthermore, by demonstrating that KBS are strongly associated with crop yield, with opposite effects observed in resistant and susceptible cultivars, we suggest that the influence of key microbial taxa is not universal but genotype‐dependent. However, we acknowledge that these findings are primarily based on statistical associations; therefore, future research utilizing synthetic communities (SynComs) and multi‐omics approaches in strictly controlled environments is needed to mechanistically validate these interactions. Altogether, these findings not only highlight the ecological importance of host genotype in determining foliar microbiome functionality, but also offer implications for breeding programs and microbiome‐based interventions. Incorporating phyllosphere‐targeted strategies, such as selecting genotypes that preferentially enrich beneficial microbes, could complement existing soil‐focused approaches and contribute to more sustainable, low‐input crop production systems.

## Conflicts of Interest

The authors declare no conflict of interest.

## Data Availability

Raw sequencing data have been deposited in the NCBI Sequence Read Archive (SRA) with the accession numbers PRJNA1290647 (fungi; SUB15457246) and PRJNA1290627 (bacteria; SUB15456590).
